# Differential action of glucocorticoids on apolipoprotein E gene expression in macrophages and hepatocytes

**DOI:** 10.1371/journal.pone.0174078

**Published:** 2017-03-29

**Authors:** Violeta Georgeta Trusca, Elena Valeria Fuior, Ioana Madalina Fenyo, Dimitris Kardassis, Maya Simionescu, Anca Violeta Gafencu

**Affiliations:** 1 Department of Genomics, Transcriptomics and Molecular Therapies, Institute of Cellular Biology and Pathology “Nicolae Simionescu” of the Romanian Academy, Bucharest, Romania; 2 Department of Basic Sciences, University of Crete Medical School, Heraklion, Crete, Greece; 3 Institute of Molecular Biology and Biotechnology, Foundation for Research and Technology of Hellas, Heraklion, Crete, Greece; Universitat des Saarlandes, GERMANY

## Abstract

Apolipoprotein E (apoE) has anti-atherosclerotic properties, being involved in the transport and clearance of cholesterol-rich lipoproteins as well as in cholesterol efflux from cells. We hypothesized that glucocorticoids may exert anti-inflammatory properties by increasing the level of macrophage-derived apoE. Our data showed that glucocorticoids increased apoE expression in macrophages in vitro as well as in vivo. Dexamethasone increased ~6 fold apoE mRNA levels in cultured peritoneal macrophages and RAW 264.7 cells. Administered to C57BL/6J mice, dexamethasone induced a two-fold increase in apoE expression in peritoneal macrophages. By contrast, glucocorticoids did not influence apoE expression in hepatocytes, in vitro and in vivo. Moreover, dexamethasone enhanced apoE promoter transcriptional activity in RAW 264.7 macrophages, but not in HepG2 cells, as tested by transient transfections. Analysis of apoE proximal promoter deletion mutants, complemented by protein-DNA interaction assays demonstrated the functionality of a putative glucocorticoid receptors (GR) binding site predicted by in silico analysis in the -111/-104 region of the human apoE promoter. In hepatocytes, GR can bind to their specific site within apoE promoter but are not able to modulate the gene expression. The modulatory blockade in hepatocytes is a consequence of partial involvement of transcription factors and other signaling molecules activated through MEK1/2 and PLA2/PLC pathways. In conclusion, our study indicates that glucocorticoids (1) differentially target apoE gene expression; (2) induce a significant increase in apoE level specifically in macrophages. The local increase of apoE gene expression in macrophages at the level of the atheromatous plaque may have therapeutic implications in atherosclerosis.

## Introduction

Apolipoprotein E (apoE) is a 35 kDa glycoprotein which has an essential contribution to the lipoprotein metabolism [1]. ApoE deficiency in humans or animal models is associated with hyperlipoproteinemia and premature atherosclerosis [2–4]. ApoE is mainly synthesized by the liver, being a component of most lipoprotein subclasses, participating in the clearance of atherogenic lipoproteins and lipoprotein remnants from the plasma. In the vascular wall, apoE secreted by macrophages is involved in the cholesterol efflux from the atheromatous plaques [5]. Bone marrow transplantation studies revealed the importance of macrophage–secreted apoE in the protection against atherosclerosis. C57BL/6J mice transplanted with bone marrow from apoE-deficient mice displayed increased atherosclerosis [6], while apoE-deficient mice receiving wild-type murine bone marrow cells displayed reduced atherosclerosis and lower hypercholesterolemia [7]. Furthermore, mice expressing apoE only in macrophages did not develop the disease despite plasma apoE level was low [8]. Using an apoE-expressing retrovirus, it was demonstrated that apoE produced by the arterial macrophages exerted beneficial effects during the early stages of atherogenesis [9]. Moreover, it was reported that macrophage-specific apoE expression was necessary for macrophage reverse cholesterol transport *in vivo*, while no significant role in this process was attributed to systemic apoE [10].

The transcription regulation of apoE gene takes place in a cell-specific manner and involves the interaction of various transcription factors with the proximal and distal regulatory elements, as recently reviewed [11]. The two hepatic control regions, HCR.1 and HCR.2, essential for the expression of the apoE/apoCI/apoCIV/apoCII gene cluster in the liver were identified by Taylor et al. [12, 13]. Subsequently, two multienhancer regions, namely ME.1 and ME.2, were found to regulate apoE expression in macrophages and adipose tissue [14]. Previous studies showed that apoE gene transcription increased upon monocyte differentiation into macrophages [15, 16]. On the other hand, the inflammatory conditions (such as those induced by lipopolysaccharides) lead to a decrease in apoE expression in macrophages [17], a process with negative consequences on cholesterol efflux from the atherosclerotic plaque. The glucocorticoids would improve the apoE level in the plaque, reversing the effect of local factors. Besides the acceleration of the cholesterol efflux, apoE secreted by macrophages at the level of the atherosclerotic plaque plays an important role in changing the macrophage phenotype from pro-inflammatory M1 to anti-inflammatory M2 [18]. This would lead to major changes in chemokines secretion at the level of the plaque and thus, to stabilization and/or a possible regression of the atheroma. Considering the multiple positive effects of apoE against atherosclerosis, various strategies to increase its expression were designed. Since systemic overexpression of apoE in mice causes hypertriglyceridemia [19], novel anti-atherosclerotic therapies which specifically target apoE expression in certain cell types are urgently needed.

Glucocorticoids play an essential role in the response to stress and the restoration of a homeostatic state [20]. The physiological and pharmacological actions of glucocorticoids are mediated by the ubiquitously expressed glucocorticoid receptors (GR), also named NR3C1 [21]. Upon glucocorticoid binding, GR are translocated to the nucleus where they modulate (induce or repress) the transcription of target genes, mainly of those involved in inflammatory and immune responses [22]. Due to the marked anti-inflammatory action of glucocorticoids recognized long time ago, these hormones are thoroughly explored as potential therapeutic drugs for the treatment of several diseases [23]. In the context of atherosclerosis- associated inflammation, glucocorticoids were reported by several studies to exert beneficial effects [24]. However, the development of novel strategies to prevent the undesirable systemic effects of glucocorticoids is still needed. Thus, targeting GR in a cell-specific manner may be an alternative of utmost importance.

Our starting hypothesis was that glucocorticoids exert their beneficial effects through an increase in macrophage-derived apoE. Herein we report that dexamethasone enhanced apoE gene expression in macrophages, but not in hepatocytes. This differential effect of glucocorticoids on apoE transcription in the two cell types prompted us to search for the regulatory elements involved in apoE gene modulation and to elucidate the mechanisms involved. Taken together, our results reveal a specific glucocorticoid-induced increase in apoE gene expression in macrophages which may have therapeutic implications in atherosclerosis.

## Material and methods

### Chemicals

Dexamethasone, Mifepristone, U0126, U-73122, PMA (phorbol myristate acetate) and o-nitrophenyl β-D-galactopyranoside were from Sigma-Aldrich (St. Louis, MO, USA). GoTaq DNA polymerase, Luciferase Assay System and Renilla Luciferase Assay System were obtained from Promega (Madison, WI, USA). FastDigest restriction enzymes were from Thermo Scientific. DMEM, RPMI-1640 and fetal calf serum were purchased from EuroClone (Milano, Italy) and Super Signal West Pico chemiluminescent substrate was from Pierce (Rockford, USA). Thioglycollate medium was obtained from HiMedia (Mumbai, India). Liberase research grade purified enzyme (05401119001) was purchased from Roche. High-Capacity cDNA reverse transcription kit, TaqMan probes for apoE (Mm01307193_g1) and actin (Mm00607939_s1) were from Applied Biosystems. TRIzol reagent was purchased from Invitrogen Life Technologies (Carlsbad, CA, USA), InnuPREP RNA Mini kit was from Analytic Jena AG and Dynabeads M-280 streptavidin magnetic beads were from Invitrogen Dynal (Oslo, Norway). Human and mouse primers for GRα and biotinylated oligonucleotides, containing a region of apoE proximal promoter or a validated sequence for NF-κB binding, were from Microsynth AG (Balgach, Switzerland), while biotinylated oligonucleotides for GRα binding were provided by VBC-Genomics (Vienna, Austria). Nuclear extract kit was purchased from Active Motif (Rixensart, Belgium). The following antibodies were used: human apoE antibody (cat no. 18171) from Immuno-Biological Laboratories Co., LTD (Takasaki, Japan), mouse apoE (cat no. BP2046) antibody from Acris Antibodies GmbH (Herford, Germany), anti-GR antibodies (sc-8992 and sc-393232) and anti-β-actin (sc-47778) antibody from Santa Cruz Biotechnology (Santa Cruz, CA). Midori Green was from Nippon Genetics Europe (Germany).

### Plasmid constructions

The apoE proximal promoter [-500/+73]apoE-luc and its deletion mutants: [-200/+73]apoE-luc, [-100/+73]apoE-luc and [-55/+73]apoE-luc, previously described [16, 17], were cloned in KpnI/XhoI sites in pGL4 vector (Promega). GR_synthRE (S900015) reporter construct (which contains a synthetic glucocorticoid response element) was purchased from SwitchGear Genomics (Belgium), and the pcDNA3-hGRα plasmid was kindly provided by Dr. Russcher (University Medical Center, Rotterdam).

### Cell culture

Cell lines RAW 264.7 murine macrophages, HepG2 human hepatocytes and HEK-293 (human embryonic kidney) were from ATCC (USA). Cells were grown in DMEM with 10% fetal calf serum and penicillin/streptomycin with 1‰ glucose for RAW 264.7 and HEK-293 cells and 4.5‰ glucose for HepG2 cells. THP-1 human monocytes were grown in RPMI-1640 supplemented with 10% heat-inactivated serum and differentiated into macrophages by exposure to PMA (50 nM for 72 hours). Mouse peritoneal macrophages (MPM) were isolated from C57BL/6J mice using thioglycollate medium, as previously reported [25]. Murine primary hepatocytes (MPH) were isolated from C57BL/6J mice using liberase research grade purified enzyme (Roche).

### Animal experimentation

Male C57BL/6J wild-type mice (Charles River Laboratories) fed standard rodent diet and water *ad libitum* were grown alternating 12 hour cycles of light and dark. Mice (10-week old) were randomly separated into two experimental groups treated as follows: i) dexamethasone-treated group received 100 μL/ mouse/ day dexamethasone (30mg/kg body weight) by gavage (n = 5 animals); ii) control group received 100 μL/ mouse/ day water by gavage (n = 6 animals). After five days of treatment, the levels of apoE gene expression in thioglycollate-elicited mouse peritoneal macrophages and in liver extracts were determined by Real-Time PCR. Animal experiments were conducted in accordance with the Directive 2010/63/EU and the European Convention for the Protection of Vertebrate Animals used for Experimental and Other Scientific Purposes; experimental protocols were approved by the Ethical Committee of the Institute of Cellular Biology and Pathology “Nicolae Simionescu”.

### RT-PCR and real-time PCR

Total cellular RNA was isolated using TRIzol reagent (from cells) and InnuPREP RNA Mini kit (from tissues); reverse transcription was performed with High-Capacity cDNA reverse transcription kit (Applied Biosystems). For RT-PCR, specific primers presented in Table 1 were used. The length of the corresponding PCR products was as follows: 389 bp (mouse apoE), 584 bp (human apoE), 294 bp (mouse GR), 368 bp (human GR), and 452 bp (human/mouse GAPDH). Real-Time PCR experiments were done using TaqMan probes and specific primers for apoE and actin genes in duplex reaction, with Universal Master Mix on a 7900 HT Applied Biosystems machine (Applied Biosystems, USA). ApoE levels were normalized to β-actin expression.

**Table 1:** The sequences of the primers used for RT-PCR, DNA pull-down assays and chromatin immunoprecipitation (ChIP) experiments.

Primers	Forward	Reverse
**RT-PCR**		
**Human apoE**	5’-CCAGCGGAGGTGAAGGAC	5’-CGCTTCTGCAG GTCATCG
**Mouse apoE**	5’-ACAGATCAGCTCGAGTGGCAAA	5’-ATCTTGCGCAGGTGTGTGGAGA
**Human GR**	5’-AACAGCAACAACAGGACCACC	5’-CCAGGGTAGGGGTGAGTTGTG
**Mouse GR**	5’-TGGAAACCTGCTATGCTTTGCTC	5’-AACCGCTGCCAATTCTGACTG
**GAPDH**	5’-ACCACAGTCCATGCCATCAC	5’-TCCACCACCCTGTTGCTGTA
**DNA pull-down assays**		
**oligonucleotides apoE (-115/-75)**	5’-Biotin-GGGGGAGAACAGCCCACCTCG TGACTGGGGGCTGGCCCA	5’-TGGGCCAGCCCCCAGTCACGAGG TGGGCTGTTCTCCCCC
**Non-specific oligonucleotides**	5’-Biotin-AGTTGAGGGGACTTTCCCAGGC	5’-GCCTGGGAAAGTCCCCTCAACT
**GR oligonucleotides**	5’-Biotin-GACCCTAGAGGATCTGTA CAGGATGTTCTAGAT	5’-ATCTAGAACATCCTGTAC AGATCCTCTAGGGTC
**GR-mut oligonucleotides**	5’-Biotin-GACCCTAGAGGATCTCAACAGGATCATCTAGAT	5’-ATCTAGATGATCCTGTTGAGATCCTCTAGGGTC
**ChIP**		
**-254/+4 apoE**	5’-GGGGTACCTCCACGCTTGGCCCCC	5’-GGGAGCTCGTGGGGCTGAGTAGGAC

### Transient transfections

RAW 264.7 macrophages, HepG2 hepatocytes, and HEK-293 cells were transiently transfected by Ca_3_(PO_4_)_2_ precipitation method [25]. Luciferase activity was assayed with the Luciferase Assay Kit from Promega and transfection efficiency was normalized to β-galactosidase activity measured using o-nitrophenyl β-D-galactopyranoside as substrate.

### DNA pull-down assays

The assays were carried out using nuclear extracts purified from regular and GRα- or cJun-overexpressing HEK-293 cells. Biotinylated oligonucleotides (corresponding to the region -115/-75 of the apoE proximal promoter, a consensus/mutated glucocorticoid response element or a non-specific sequence) immobilized on Dynabeads M-280 Streptavidin, were used as previously reported [17]. The sequence of the oligonucleotides used for DNA pull-down assays is shown in Table 1. GR proteins bound to the biotinylated oligonucleotides were detected by immunoblotting using anti-GR antibodies after protein separation by SDS-PAGE.

### Immunoblotting

Mouse peritoneal macrophages and murine primary hepatocytes were exposed to various concentrations of dexamethasone for 24 hours. Afterwards, cell lysates were subjected to SDS-PAGE and transferred onto nitrocellulose membrane. The blots were probed with rabbit anti-apoE or mouse anti-β-actin antibodies To detect the nuclear translocation of GR under dexamethasone treatment, cytoplasmic and nuclear fractions of RAW 264.7 and HepG2 cells were isolated using a commercial kit (Active Motif, Belgium). The proteins from both fractions were separated on SDS-PAGE and blotted onto nitrocellulose membrane. The blots were incubated with monoclonal anti-GR, anti-TFIID and anti-actin antibodies. After incubation with the corresponding secondary antibodies coupled with horseradish peroxidase, the protein bands were detected using Super Signal West Pico chemiluminescent substrate and quantified with Total Lab software (NonLinear Dynamics, USA).

### Chromatin immunoprecipitation

Chromatin immunoprecipitation experiments were performed as previously reported [26] using chromatin from PMA-differentiated THP1 macrophages or HepG2 hepatocytes, untreated or treated with 1 μM dexamethasone for 24 hours. Chromatin was immunoprecipitated with anti-GR antibodies and analyzed by PCR using primers presented in Table 1. PCR products were analyzed by agarose gel electrophoresis after staining with Midori Green.

### Statistics

Results represent means ± standard deviations. Statistical significance was calculated using a two-tailed t-test (GraphPad Software, San Diego, USA). Differences with p<0.05 were considered statistically significant. Symbols used in the figures are: *** (p<0.001), **(p<0.01) and *(p<0.05).

## Results

### Dexamethasone upregulates apoE expression in macrophages, but not in hepatocytes

**Fig 1:**
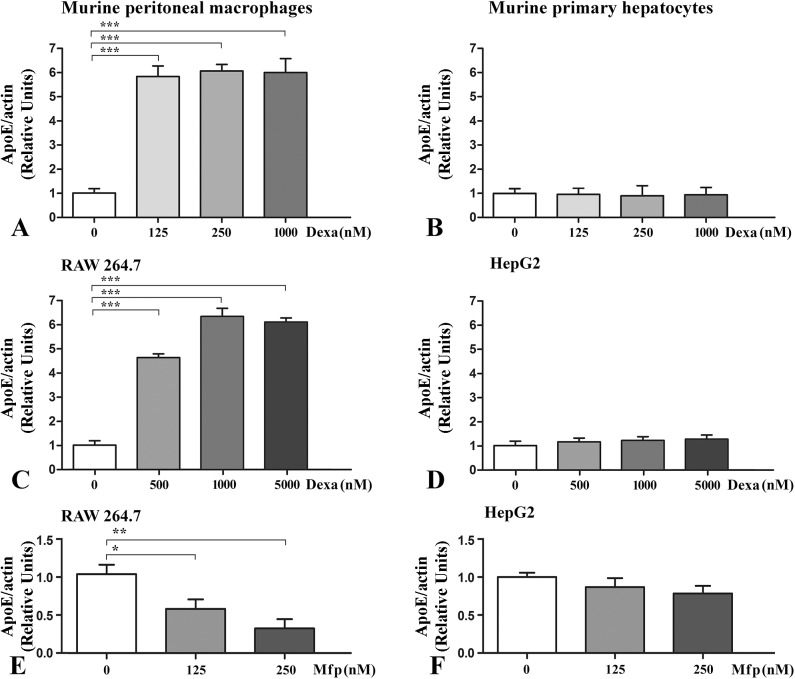
*In vitro* cell-specific modulation of apoE gene expression in response to glucocorticoid receptor agonist and antagonist in cultured macrophages and hepatocytes. ApoE gene expression in freshly isolated mouse peritoneal macrophages (A), murine primary hepatocytes (B) and established cell lines, RAW 264.7 (C, E) and HepG2 (D, F) exposed for 24 hours to increasing concentrations of dexamethasone (GR ligand) or mifepristone (GR antagonist) was assessed by Real-Time PCR. Dexamethasone increases up to ~6 fold apoE mRNA levels in mouse peritoneal macrophages (A) and RAW 264.7 macrophages (C) whereas apoE gene expression is not significantly affected by dexamethasone in murine primary hepatocytes (B) and HepG2 hepatocytes (D). Mifepristone treatment significantly decreases apoE mRNA levels in RAW 264.7 macrophages (E) but not in HepG2 hepatocytes (F). The symbols are: *** p<0.001, **p<0.01 and *p<0.05.

To address the issue of cell-specific apoE regulation in response to glucocorticoids, first we evaluated apoE gene expression in macrophages compared to hepatocytes in the presence of dexamethasone, a GR agonist. To this aim, apoE gene expression was determined by Real-Time PCR in freshly isolated mouse peritoneal macrophages and primary cultures of murine hepatocytes, as well as in established cell lines (RAW 264.7 and HepG2) exposed for 24 hours to various concentrations of dexamethasone. As illustrated in Fig 1A, a differential effect of glucocorticoids on apoE gene expression in macrophages and hepatocytes was revealed. In both mouse peritoneal macrophages and RAW 264.7 cells, treatment with dexamethasone (125–1000 nM) induced a ~6 fold increase in apoE mRNA levels (p<0.001), as compared to control cells (Fig 1A and 1C, respectively). In contrast with the results obtained in macrophages, in murine primary hepatocytes and HepG2 hepatocytes dexamethasone treatment did not induce significant changes (p>0.05) in apoE mRNA levels (Fig 1B and 1D, respectively). Treatment of macrophages and hepatocytes with mifepristone (a GR antagonist) caused a dose-dependent reduction in apoE mRNA in macrophages (Fig 1E), down to ~30% of the basal apoE level for 250 nM mifepristone (p<0.01). By contrast, mifepristone had no statistically significant effect on apoE gene expression in hepatocytes (p>0.05), as shown in Fig 1F.

**Fig 2:**
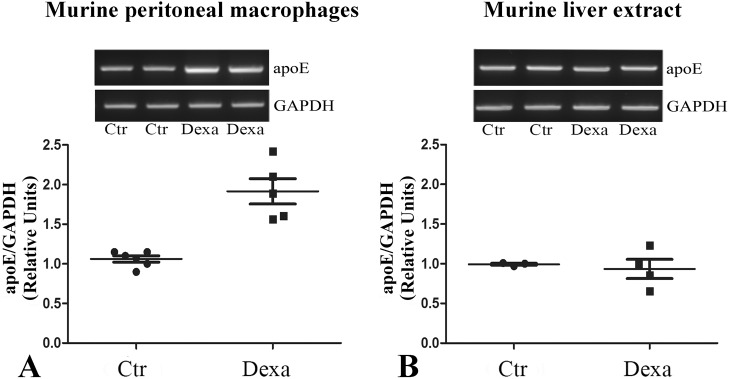
*In vivo* cell-specific effect of dexamethasone on apoE expression in macrophages and liver of C57BL/6J mice. Representative images show the expression of apoE and GAPDH genes in mouse peritoneal macrophages **(A)** and liver extracts **(B)**, as detected by RT-PCR (upper panels). Quantification of apoE gene expression normalized to GAPDH in peritoneal macrophages and liver extracts in dexamethasone-treated mice, as compared to control mice, is depicted in the lower panels. Five-day dexamethasone (30mg/kg body weight) treatment of C57BL/6J mice significantly increases apoE mRNA levels in mouse peritoneal macrophages (p<0.001), but does not affect hepatic apoE gene expression (p>0.05).

To test whether the differential *in vitro* effect of dexamethasone on the two cellular types occurs *in vivo* as well, we treated C57BL/6J mice with dexamethasone (30mg/kg body weight) by gavage (‘Dexa’ group) and compared them with the control mice (‘Ctr’ group) which received only the corresponding amount of water by the same route of administration. Dexamethasone effect on apoE gene expression in macrophages and liver was evaluated by RT-PCR. Representative images of Midori-stained agarose gels, showing apoE and GAPDH gene expression in peritoneal macrophages and liver extracts isolated from both dexamethasone-treated and control mice, are presented in Fig 2, upper panels. Quantification of apoE expression in macrophages from treated and control animals revealed that dexamethasone administration to C57BL/6J mice induced a two-fold increase (p<0.001) in apoE mRNA levels in peritoneal macrophages, whereas apoE gene expression in liver was not significantly affected (p>0.05) compared to control mice (lower panels, Fig 2A and 2B, respectively). These data corroborated and confirmed the results obtained *in vitro* regarding the differential, cell-specific effects of dexamethasone on apoE gene expression in macrophages and hepatocytes.

**Fig 3:**
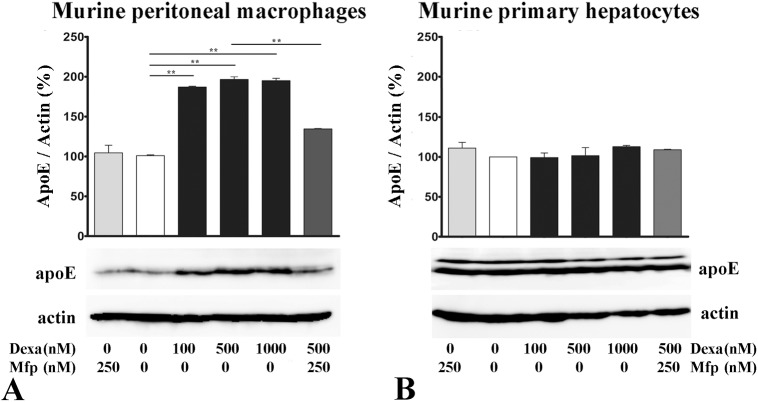
Macrophage-specific enhancement of apoE protein expression by dexamethasone. **(A)** Dexamethasone (100–1000 nM, 48 hours) significantly increases the expression of apoE protein in mouse peritoneal macrophages (MPM), and mifepristone attenuates this effect. **(B)** No alteration in apoE protein expression is detected in murine primary hepatocytes (MPH) under dexamethasone treatment. Western blots results were normalized to β-actin. Upper panels represent the quantification of three independent experiments, and lower panels are representative images. The symbol ** is for p<0.01.

We next assessed the effect of dexamethasone on apoE protein expression in mouse peritoneal macrophages (MPM) and murine primary hepatocytes (MPH). For this purpose, cells were treated with dexamethasone (100–1000 nM) or mifepristone (250 nM) for 48 hours and the cellular apoE protein levels were determined by immunoblotting. Our data showed that increasing concentrations of dexamethasone (100–1000 nM) induced a significant enhancement (~1.8 fold) in apoE protein level in mouse peritoneal macrophages (Fig 3A). The treatment with mifepristone (250 nM) attenuated the effect of dexamethasone on apoE expression in macrophages and did not affect apoE expression in the absence of dexamethasone (Fig 3A). By contrast, apoE protein level in murine primary hepatocytes was not significantly modified by dexamethasone or mifepristone treatment, the cells exhibiting similar levels in treated and control samples (Fig 3B). The expression of β-actin was used as endogenous control. Lower panels in Fig 3 illustrate representative Western blots for apoE in murine peritoneal macrophages (Fig 3A) and primary hepatocytes (Fig 3B).

### Mechanisms of macrophage-specific transactivation of apoE promoter by ligand-activated GRα

**Fig 4:**
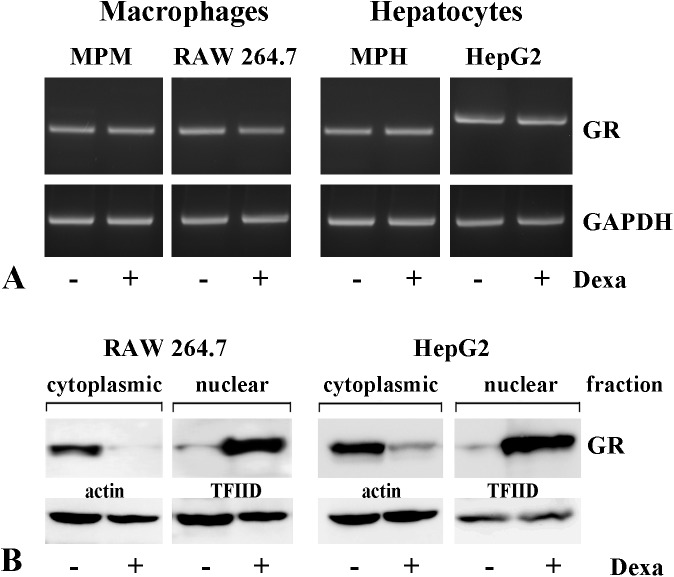
Glucocorticoid receptor (GR) expression and intracellular distribution in macrophages and hepatocytes upon dexamethasone treatment. (A) Macrophages (MPM, RAW 264.7) and hepatocytes (MPH, HepG2) express similar levels of GRs independently of dexamethasone treatment, as evaluated by RT-PCR with GAPDH used as a reference gene. (B) Treatment of RAW 264.7 macrophages and HepG2 cells with 1 μM dexamethasone for 24 hours leads to GR translocation into the cell nucleus, as detected by Western Blot; actin and TFIID were used as protein load controls for the cytoplasmic and nuclear fractions, respectively.

Having established the differential modulation of apoE by GR ligand, we further proceeded to investigate the mechanisms underlying this process. First, we tested whether glucocorticoid receptors are differentially expressed in macrophages and hepatocytes or if there is an induction of GR by dexamethasone treatment. We analyzed the expression of GRα in macrophages (MPM and RAW 264.7) and hepatocytes (MPH and HepG2) treated with dexamethasone (1 μM) as compared to untreated cells; GAPDH expression was used as endogenous control. Our data showed a similar GRα expression in the analyzed cells, regardless of dexamethasone treatment (Fig 4A).

Next, we tested the ability of dexamethasone to induce GR nuclear translocation in macrophages and hepatocytes. For this, RAW 264.7 and HepG2 cells were exposed to 1 μM dexamethasone (24 hours), and the cytoplasmic and nuclear fractions were probed for GR by immunoblotting, using actin and respectively TFIID as markers for the quality of cellular fractionation. We found that each cell type endogenously expressed GRα, and the receptors were mainly distributed in cytosolic fraction, in the absence of the ligand (Fig 4B). The decrease of GR in the cytoplasmic fraction was accompanied by a strong increase in the nuclear extracts obtained from dexamethasone–treated RAW 264.7 macrophages as well as in HepG2 cells (Fig 4B) suggesting the transport of GR from the cytoplasm to the nucleus in the presence of the ligand.

**Fig 5:**
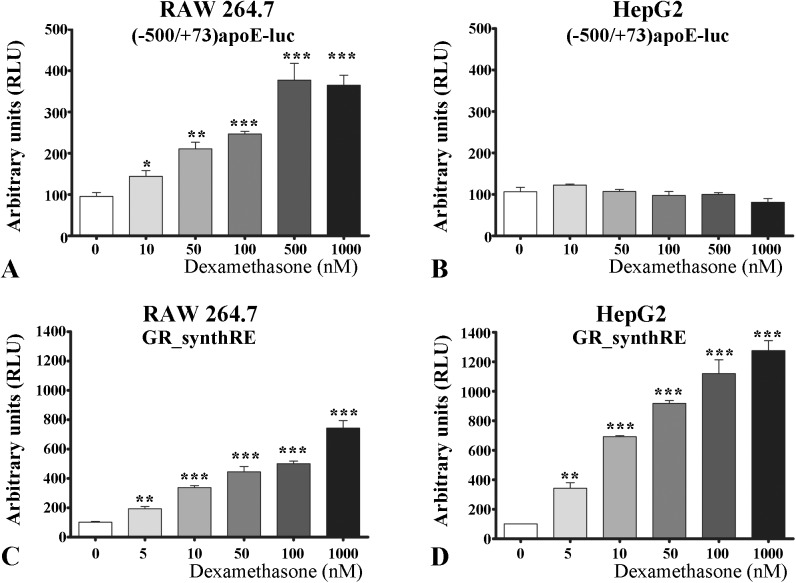
Transactivation of apoE proximal promoter by dexamethasone in macrophages. RAW 264.7 **(A, C)** and HepG2 **(B, D)** cells were transiently transfected using plasmids containing apoE proximal promoter ([-500/+73]apoE-luc) or a minimal promoter with glucocorticoid responsive elements (GR_synthRE) and subsequently treated with increasing concentrations of dexamethasone (10–1000 nM) for 24 hours. The activities of [-500/+73]apoE-luc and GR_synthRE constructs were determined by luciferase assays. In RAW 264.7 macrophages, dexamethasone enhances the activity of apoE proximal promoter in a dose-dependent manner **(A)** whereas in HepG2 hepatocytes, all tested concentrations of dexamethasone (10–1000 nM) do not significantly (p>0.05) affect apoE promoter activity **(B)**. In both macrophages **(C)** and hepatocytes **(D)**, the activity of GR_synthRE promoter is significantly raised by dexamethasone (10–1000 nM). The symbols are: *** p<0.001, **p<0.01 and *p<0.05.

To test whether apoE promoter transcriptional activity is modulated by glucocorticoids, transient transfection experiments were performed in macrophages and hepatocytes. To this aim, RAW 264.7 and HepG2 cells were transiently transfected with the [-500/+73]apoE-luc construct and then the cells were treated for 24 hours with dexamethasone at concentrations ranging from 10 nM to 1 μM. The activity of apoE promoter was evaluated based on the luciferase reporter gene and normalized to the activity of β-galactosidase, which was co-transfected. As illustrated in Fig 5A, a dose-dependent increase in apoE promoter activity by dexamethasone was recorded in RAW 264.7 macrophages. The apoE promoter activity reached a plateau (~3.7 fold increase as compared to control, p<0.001) for dexamethasone doses higher than 500 nM. In contrast to the data obtained in macrophages, in HepG2 hepatocytes the activity of apoE promoter was not modulated by dexamethasone (Fig 5B, p>0.05).

As a positive control, we used a plasmid containing the luciferase gene under the control of a minimal promoter containing glucocorticoid response elements (GR_synthRE construct). As expected, dexamethasone treatment enhanced the activity of the reporter gene in both RAW 264.7 and HepG2 cells (Fig 5C and 5D). Moreover, the data showed that dexamethasone had a similar effect on the activity of apoE promoter and GR_synthRE in macrophages (Fig 5A and 5C) and revealed a remarkable difference in the behavior of these two promoters under the action of GR ligand in hepatocytes (Fig 5B and 5D, respectively). Interestingly, when the reporter gene expression was driven by the synthetic promoter containing glucocorticoid response elements, the induction by dexamethasone was even higher in hepatocytes as compared with macrophages (Fig 5C and 5D).

**Fig 6:**
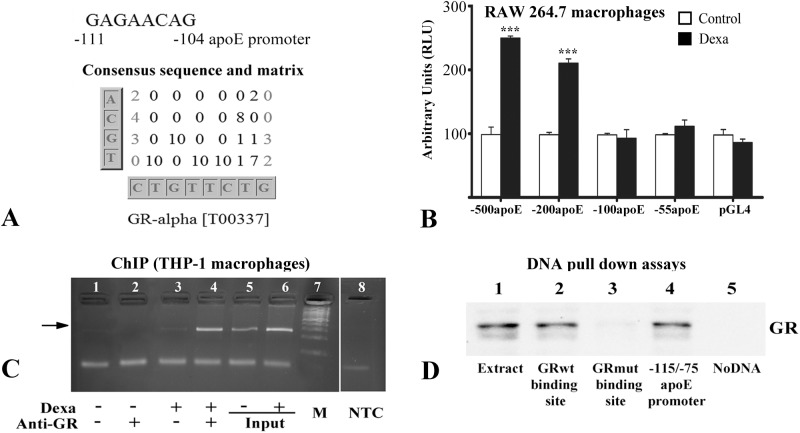
In macrophages glucocorticoid receptors (GRs) bind to a consensus sequence in apoE proximal promoter and upregulate its activity. (A) *In silico* analysis of apoE proximal promoter revealing a GRα binding site in the region -111→-104. (B) In transiently transfected RAW 264.7 macrophages, dexamethasone significantly enhances the activity of [-500/+73] apoE promoter and of the [-200/+73] fragment, but not the activities of the [-100/+73] and [-55/+73] deletion fragments or the activity of the pGL4 vector. (C) Chromatin immunoprecipitation assay shows that GR proteins are recruited to the human apoE promoter in PMA-differentiated THP1 macrophages treated with dexamethasone (lane 4). No bands were observed in untreated macrophages (lane 2) or when the antibodies were omitted (lanes 1 and 3). PCR using the input as template and primers for apoE promoter generated the expected bands (lanes 5 and 6, respectively). Lane 8 represents the no template control PCR (NTC). Specific 258 bp PCR product is indicated by the arrow. (D) GR proteins bind efficiently to oligonucleotides corresponding to the -115/-75 region of apoE promoter as revealed by DNA pull-down assays (lane 4). GR proteins also bind to control oligonucleotides containing a known glucocorticoid responsive element (lane 2), but do not bind to the mutated glucocorticoid responsive element (lane 3). Whole cell extract was used as positive control (lane 1); there is no binding of GRs to uncoupled Dynabeads, used as negative control (lane 5). The symbol *** is for p<0.001.

To identify the region of the apoE promoter required for transactivation by glucocorticoids, we searched for putative GR binding sites. Sequence analysis by *Matrix Transcription Factors Site* (http://alggen.lsi.upc.es/) predicted the presence of a GRα binding site in the -111→-104 region of the human apoE proximal promoter. Fig 6A presents the region of apoE promoter containing the predicted GRα site, the consensus sequence and the matrix provided by this analysis. Consequently, we investigated whether this putative GRα binding site on human apoE promoter is functional and responsible for the induction of apoE gene expression by dexamethasone in macrophages. For this, transient transfection experiments in RAW 264.7 macrophages were performed using constructs containing human apoE proximal promoter or its deletion fragments. As illustrated in Fig 6B, dexamethasone significantly increased the activity of the proximal apoE promoter (-500apoE) and of its deletion fragment -200apoE (p<0.001), but did not enhance the activities of -100apoE and -55apoE promoter fragments and of the pGL4 vector, used as a control (p>0.05). These data indicated that the region of apoE proximal promoter required for dexamethasone-induced transactivation is located in the region -200 to -100 in the human apoE gene.

To test the *in vivo* recruitment of GRs to the apoE promoter we performed chromatin immunoprecipitation assays, using PMA-differentiated THP-1 human macrophages. For this, the cross-linked chromatin was immunoprecipitated using anti-GR antibodies; PCR was performed using primers that amplified the region -254/+4 of the human apoE promoter (presented in Table 1). The results illustrated in Fig 6 indicated that GR proteins were recruited to apoE promoter in dexamethasone-treated PMA-differentiated THP-1 macrophages (Fig 6C, lane 4). No binding of GRs was found in cells that were not exposed to dexamethasone (Fig 6C, lane 2) or when the antibodies were omitted in chromatin immunoprecipitation using untreated or dexamethasone-treated cells (Fig 6C, lanes 1 and 3, respectively). PCR using the input prepared from treated or untreated cells as template resulted in the expected bands (Fig 6C, lanes 5 and 6). The arrow in Fig 6C indicates the specific band of the apoE promoter fragment (258 bp), that is absent in the ‘no template control’ reaction (lane 8, NTC). The DNA ladder (100 bp ladder, from ThermoScientific) is represented in Fig 6C, lane 7 (M).

*In vitro* binding of GR to apoE promoter was tested by DNA pull down experiments, using biotinylated oligonucleotides corresponding to the -115/-75 region of the human apoE promoter and whole cell extracts prepared from HEK-293 cells. The results showed that GR proteins bound efficiently to the -115/-75 region of apoE promoter (Fig 6D, lane 4). To validate the DNA binding experiments, we tested GR binding to control oligonucleotides containing a well-characterized glucocorticoid responsive element (whose sequence is given in Table 1). Thus, GR was found to bind oligonucleotides corresponding to glucocorticoid binding site (Fig 6D, lane 2), while no GR binding occurred when the oligonucleotides contained a mutated glucocorticoid responsive element (Fig 6D, lane 3). Whole cell extract served as a positive control (Fig 6D, lane 1); no binding of GR was detected in uncoupled Dynabeads used as negative control (Fig 6D, lane 5).

### Molecular impairment of apoE induction by glucocorticoids in hepatocytes

**Fig 7:**
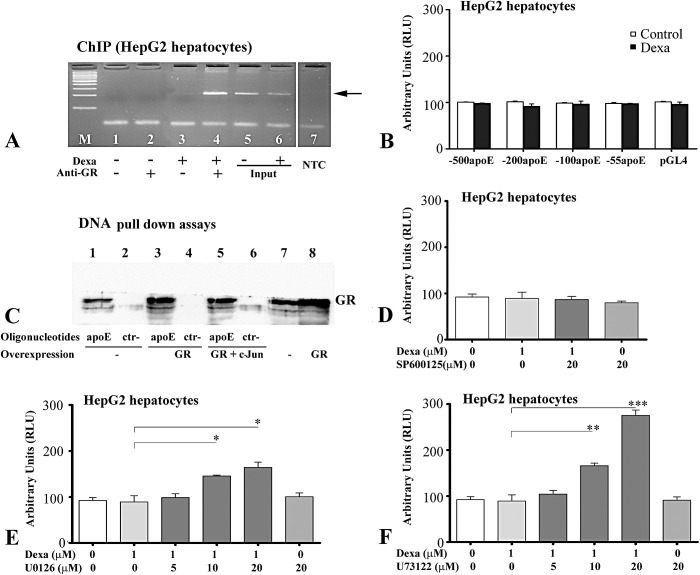
In hepatocytes, glucocorticoid receptors (GRs) bind to apoE proximal promoter but are not capable to regulate apoE gene expression due to the interaction with other signaling pathways. (A) Dexamethasone induces GR recruitment to apoE promoter in HepG2 hepatocytes as revealed by ChIP assay (lane 4). In the absence of the ligand, GRs do not bind to apoE promoter (lane 2). In controls, the chromatin of dexamethasone-treated or untreated cells was processed omitting the antibodies (lanes 3 and 1, respectively). PCR using the input prepared from treated or untreated cells as template produced the expected bands (lanes 5 and 6, respectively). The arrow indicates the specific 258 bp band corresponding to apoE promoter, that is absent in the no template control reaction (lane 7, NTC). In lane 1 (M), marker DNA ladder is shown. (B) In transiently transfected HepG2 cells, exposure to dexamethasone does not significantly enhance the activity of [-500/+73] apoE promoter, its deletion fragments or pGL4 vector, used as a control (p>0.05). (C) DNA pull down experiments show that GR bind to the -115/-75 apoE promoter region in untransfected cells (lane 1) and binding is enhanced by overexpression of GRα (lane 3). A partial (~30%) inhibition of GR binding was detected in cells overexpressing both GR and c-Jun (lane 5). No binding occurs to non-specific oligonucleotides when the cells express only endogenous GR (lane 2), overexpress GR (lane 4) or overexpress both GR and c-Jun (lane 6). Western blots probed for GRα of whole extracts prepared from cells expressing only the endogenous protein or overexpressing it are illustrated in lanes 7 and 8, respectively. (D-F) The effects of various inhibitors on dexamethasone-treated HepG2 cells transiently transfected with plasmids containing luciferase under apoE proximal promoter. (D) Dexamethasone does not significantly modulate apoE promoter activity in the presence or absence of the JNK inhibitor, SP600125 (E) Exposure of HepG2 cells to dexamethasone (1 μM, for 24 hours) in the presence of increasing concentrations of MEK1/2 inhibitor (U0126) induces a dose-dependent enhancement of apoE promoter activity (F). Dexamethasone treatment in the presence of PLA2/PLC inhibitor U73122 increases apoE promoter activity as a function of the inhibitor concentration. The symbols are: *** p<0.001, **p<0.01 and *p<0.05.

We hypothesized that the lack of modulation of apoE gene expression by glucocorticoids in hepatocytes was due to the fact that GR does not bind to apoE promoter in this cell type and we tested this hypothesis by chromatin immunoprecipitation assays. Surprisingly, ChIP experiments performed using HepG2 hepatocytes indicated that dexamethasone induced GR binding to apoE promoter (Fig 7A, lane 4), but that in the absence of the ligand, GR did not bind to apoE promoter (Fig 7A, lane 2). The negative controls using samples prepared from dexamethasone-treated or untreated cells, in which the antibodies were omitted, as well as the amplification without template (NTC) resulted in no PCR products (Fig 7A, lanes 1, 3 and 7, respectively). The input (1% from starting sheared chromatin) for ChIP assay obtained from untreated or dexamethasone-treated hepatocytes produced the expected 258 bp band (Fig 7A, lane 5 and 6, respectively).

Next, we investigated whether apoE promoter contains negative regulatory regions responsible for hepatocyte-specific inhibition of glucocorticoid action. For this, transient transfections experiments were performed in HepG2 hepatocytes using constructs containing apoE proximal promoter (-500apoE) or its deletion fragments (-200apoE, -100apoE and -55apoE). Eighteen hours later, the medium was replaced and the cells were treated with 1 μM dexamethasone for 24 hours. Then, the activity of luciferase reporter gene was measured and normalized to β-galactosidase activity. As shown in Fig 7B, the activity of apoE promoter (-500apoE), of its deletion fragments and of pGL4 vector (used as a control) was not influenced by dexamethasone (p>0.05).

Considering that additional cofactors could be involved in the cell-specificity action of GR, we searched for other transcription factors that may influence GR binding on apoE promoter or its activation. Since a c-Jun binding site was found to be located adjacent to GR binding site in apoE promoter, we tested whether c-Jun competes with GR binding to this region, impairing apoE gene induction by glucocorticoids in hepatocytes. To this aim, we performed DNA pull down experiments using biotinylated oligonucleotides containing -115/-75 apoE promoter region and extracts prepared from HEK-293 cells, untransfected or overexpressing GRα alone or together with c-Jun. The results of these experiments showed that GR bound to the -115/-75 region when GR was overexpressed (Fig 7C, lane 3). As expected, whole cell extract containing only the endogenous GR generated a lower signal (Fig 7C, lane 1). Interestingly, a partial inhibition of GR binding was noticed when cells overexpressed both GRα and c-Jun (Fig 7C, lane 5). In the corresponding negative controls, no GR binding was detected to non-specific oligonucleotides (shown in Table 1) whether whole cell extracts were prepared from cells overexpressing GRα alone (Fig 7C, lane 4), cells overexpressing both GRα and c-Jun (Fig 7C, lane 6) or from untransfected cells (Fig 7C, lane 2). The levels of endogenous and overexpressed GRα in the whole cell extracts used for DNA binding experiments were confirmed by Western blot (Fig 7C, lane 7 and 8, respectively).

To test whether c-Jun phosphorylation influences apoE gene modulation by glucocorticoids in hepatocytes, transient transfection experiments in HepG2 hepatocytes were performed using constructs containing proximal -500apoE promoter. Eighteen hours post- transfection, cells were treated with dexamethasone (1 μM), JNK inhibitor (SP600125, 20 μM) or both. As shown in Fig 7D, dexamethasone did not modulate the apoE promoter activity in the presence or in the absence of SP600125.

Further, we assessed whether MEK1/2 and PLC or PLA2 pathways might interfere with apoE gene modulation by glucocorticoids in hepatocytes. Transient transfections experiments showed that the treatment of HepG2 cells with 1 μM dexamethasone in the presence of increasing amounts of U0126 (MEK1/2 inhibitor) induced a dose-dependent increase of apoE promoter activity detectable at 10 and 20 μM (Fig 7E, columns 4 and 5), but not at 5 μM U0126 (Fig 7E, column 3). The highest concentration of inhibitor (20 μM) used alone did not alter apoE promoter activity (Fig 7E, last column).

When HepG2 cells transiently transfected with the luciferase gene under the control of apoE proximal promoter were treated with dexamethasone and increasing concentrations of U73122 (a PLC and PLA2 inhibitor), a dose-dependent enhancement in apoE promoter activity was observed (Fig 7F), attaining ~3 fold increase at 20 μM.

It is worth to mention that in macrophages none of the above mentioned inhibitors (U0126, U73122, and SP600125) affected the upregulatory effect of dexamethasone on apoE promoter activity (data not shown).

Taken together, these results indicate that in hepatocytes ligand-activated glucocorticoid receptors bind to the apoE proximal promoter, but the biological functionality is impaired by other factors activated through MEK1/2 and PLA2/PLC.

## Discussion

Apolipoprotein E has an anti-atherosclerotic action, being involved in the transport and clearance of circulating, cholesterol-rich lipoproteins, as well as in the cholesterol efflux from cells [27]. Within the atherosclerotic plaque, macrophage-derived apoE controls the evolution of the lesion not only by influencing cholesterol levels, but also through other pleiotropic effects [28]. We have previously reported that pro-inflammatory factors suppress apoE expression in macrophages, by a mechanism involving the action of AP1 and NF-κB transcription factors on apoE proximal promoter [15]. However, the mechanisms responsible for the modulatory effect of anti-inflammatory factors on apoE gene expression are not entirely elucidated.

Glucocorticoids are known for their anti-inflammatory and immunosuppressive properties, for which they have been used for decades in the treatment of several diseases [20, 29]. The beneficial effects of glucocorticoids are believed to be mainly attributable to the induction of the expression of anti-inflammatory proteins, as well as to the inhibition of various pro-inflammatory genes [30]. Among the multitude of synthetic glucocorticoids that have been developed for therapeutic use, dexamethasone is the most commonly used in humans. The effect of dexamethasone on atherogenesis has been investigated in both humans and animal models. Low doses of dexamethasone increase human plasma HDL-cholesterol concentrations and the size of HDL particles, by mechanisms that are not elucidated, yet [31]. In rabbits fed a cholesterol-rich diet, intramuscular injection of dexamethasone inhibited aortic atherosclerosis, though it aggravated hyperlipidemia [32]. The authors proposed that the anti-atherogenic effect of dexamethasone was due to the inhibition of the accumulation of macrophages and atherogenic lipoproteins into the aortic intima [33]. In hypercholesterolemic ApoE3 Leiden mice, short-term treatment with dexamethasone significantly reduced vein graft thickening by a mechanism involving a decreased expression of pro-inflammatory cytokines [34]. By contrast, in the absence of apoE, dexamethasone treatment of HFD mice did not significantly affect the size of the atherosclerotic lesions [35]. Taken together, these data may highlight the importance of dexamethasone-induced apoE in macrophages. Co-treatment for long periods with pitavastatin and dexamethasone may have a harmful effect, leading to an increase in the atherosclerotic lesions size, process mediated by FABP4. Validation of this synergic effect of statins and dexamethasone in other atherosclerosis models, such as the LDLR deficient mouse, may provide an indication regarding the need of precaution in the clinical administration of statins together with dexamethasone.

Most glucocorticoid responses are exerted via the intracellular glucocorticoid receptors, ligand-dependent transcription factors which belong to the nuclear receptor superfamily [21]. Since GRs can be found in almost all the tissues, it was speculated that glucocorticoids could affect nearly all cells in the body [36]. However, the specificity of the transcriptional regulation by glucocorticoids is based on the occurrence of the ligand, the level and the isoform of the GR as well as the recruitment of certain cofactors and corepressors [37]. Numerous target genes are positively or negatively regulated by GRs. Interestingly, apoAI and apoAII, two major protein constituents of HDL, are differentially regulated by dexamethasone, with the first having its hepatic expression elevated and the latter decreased [38]. Scavenger receptor class B type I was found to be down-regulated by glucocorticoids in adrenal and ovarian cells [39].

The key finding of our study is that dexamethasone differentially modulates apoE in macrophages and hepatocytes, two cell types that both synthesize and secrete apoE. The specific modulation in macrophages is important since it promotes peripheral apoE expression, avoiding the increased levels of systemic apoE provided by the hepatocytes, which may lead to hypertriglyceridemia [19]. Our experiments revealed that dexamethasone treatment of mouse peritoneal macrophages and RAW 264.7 macrophages significantly increased apoE, at both mRNA and protein levels (Figs 1A, 1C, 2A and 3A) while treatment with RU-486 (a GR antagonist) of RAW 264.7 macrophages decreased apoE gene expression (Fig 1E). Surprisingly, mifepristone decreased apoE mRNA levels in RAW 264.7 macrophages, but had no significant effect on apoE protein level in primary macrophages. The constant level of the protein in the presence or absence of mifepristone may be due to the ability of apoE to be recycled, avoiding lysosomal degradation [40]. Thus, the mRNA level may be decreased during Mfp treatment, but this is not reflected in the protein level, which remains constant. These data extend the results obtained by Zuckerman et al. showing that dexamethasone induced a 2–4 fold enhancement in apoE mRNA level and protein secretion in peritoneal macrophages as well as in macrophage-derived foam cells [41]. The data regarding dexamethasone-induced apoE gene upregulation in macrophages corroborate with the reported inhibitory effect of free- or liposome encapsulated- dexamethasone on cellular cholesterol accumulation in macrophages or foam cells [42].

In contrast, in murine primary hepatocytes and HepG2 hepatocytes apoE mRNA levels were not significantly affected by *in vitro* treatment with GR agonist or antagonist (Fig 1D and 1F) or by *in vivo* administration of dexamethasone (Fig 2B). Our results showing the lack of hepatic apoE regulation by dexamethasone in both murine primary hepatocytes and HepG2 cells do not diverge significantly from the results of Staels and co-workers, who found that apoE gene expression in rat liver was only slightly decreased after dexamethasone administration [38]. However, another study reported that in cultured fat-storing Ito cells from rat liver, apoE expression was reduced 80% by dexamethasone [43].

Despite decades of studies regarding the molecular mechanisms of GR action, the mechanisms underlying the tissue-specific effects of glucocorticoids are still poorly understood. Considering the differential modulation of apoE expression induced by dexamethasone, we investigated the glucocorticoid-induced cell-specific mechanism of apoE gene regulation in macrophages and hepatocytes. Firstly, we verified whether this effect was due to a difference in the basal GR level in the two cell types and if dexamethasone was effective in promoting GR translocation into the nucleus, as expected. Our results showed a similar level of GRs in macrophages and hepatocytes (in primary cultures or in established cell lines), as assessed by RT-PCR (Fig 4A); moreover, the cells exposed to dexamethasone had significantly decreased cytoplasmic GR levels, with subsequent GRs translocation into the cell nucleus (Fig 4B).

In an attempt to understand the mechanisms by which glucocorticoids regulate apoE expression in macrophages and hepatocytes, we tested if dexamethasone could promote transactivation of apoE proximal promoter. Dexamethasone significantly enhanced the transcriptional activity of apoE promoter in macrophages (Fig 5A), but not in hepatocytes (Fig 5B), as shown by transient transfection experiments. In macrophages, the induction of apoE promoter activity was detected at a concentration as low as 10 nM dexamethasone (Fig 5A); a similar concentration was previously reported to induce hepatic apoAI gene expression [38]. Interestingly, despite the fact that in hepatocytes apoE promoter activity could not be induced by dexamethasone, the synthetic promoter containing GR binding sites was dose-dependently transactivated by the same ligand, suggesting that other regulatory elements within apoE promoter play a role in this transcriptional modulation.

To gain further insight into the mechanism of apoE gene regulation by glucocorticoids, we tested whether active GR binding sites are present in apoE proximal promoter. Multiple experimental approaches, such as transient transfections, ChIP and DNAP assays were employed to test the functionality of the putative GR binding site identified in the -111→ -104 region of the human apoE proximal promoter by *in silico* analysis. The results confirmed that dexamethasone-activated GR directly interacted with apoE proximal promoter and up-regulated apoE gene expression in macrophages (Fig 6). Since the transactivation effect exerted by glucocorticoids on apoE promoter is reflected in apoE mRNA and protein expression, we did not include in our study the distal regulatory elements of apoE gene, such as the multienhancers.

In contrast with the results obtained in macrophages, in hepatocytes no apoE modulation occurred, despite GR binding as revealed by ChIP experiments (Fig 7A). The cell-specificity of GR-induced apoE modulation resides in the involvement of additional cofactors, since only a small proportion of GR target genes are directly regulated through conventional glucocorticoid response elements, while the majority of the genes are regulated by glucocorticoids via composite glucocorticoid-responsive regions, in which additional transcription factors participate [44].

In different cellular systems, GR can also interfere with signalling pathways or modulate the activity of various transcription factors, among which the most important are proinflammatory molecules such as MEK kinases, phospholipases, AP-1 complex, NF-κB, STAT and NF-AT [44, 45]. Due to the location of a consensus sequence for c-Jun adjacent to GR binding site, first we searched whether the ability of GR to induce apoE gene in hepatocytes is impaired by c-Jun binding. The results of DNA pull down assays showed that GR binding to apoE promoter was not abrogated by c-Jun overexpression, but was just slightly (~30%) diminished (Fig 7C and 7D). Thus, we concluded that c-Jun is just one of the factors that impair the modulatory effect glucocorticoids have on apoE. Moreover, our experiments revealed the involvement of MEK1/2 and of PLA2/PLC pathways in the modulation of apoE promoter activity by glucocorticoids, only in hepatocytes (Fig 7E and 7F). Our data showed that a combinatorial effect of different pathways leads to the inability of glucocorticoids to modulate apoE expression in hepatocytes.

**Fig 8:**
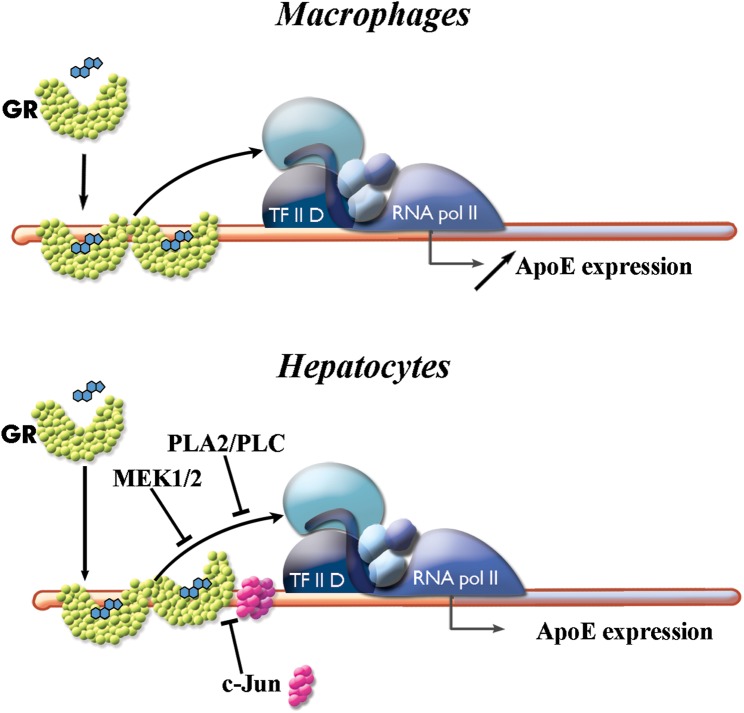
Schematic representation of cell-specific modulation of apoE gene expression by ligand-activated glucocorticoid receptors (GRs). In macrophages, ligand-activated GR binds to its specific site located on apoE proximal promoter leading to an increase in apoE promoter activity and enhancing apoE transcription. In hepatocytes, despite the direct interaction between dexamethasone-activated GR and apoE promoter, GR cannot modulate apoE transcription. The binding of c-Jun on its specific site located next to the GR binding site inhibits only partially GR binding. MEK1/2 and PLA2/PLC signaling also contributes to the inhibition of GR action on apoE gene.

In conclusion, we revealed that glucocorticoids differentially target apoE gene expression, increasing its level specifically in macrophages (Fig 8). These data highlight the potential therapeutic application of these anti-inflammatory agents that by locally inducing apoE gene expression may participate in the enhanced cholesterol efflux or provide other beneficial effects.
